# Impact of COVID-19 pandemic on mental health and health behaviors in Swedish adolescents

**DOI:** 10.1177/14034948211021724

**Published:** 2021-06-08

**Authors:** Yun Chen, Walter Osika, Göran Henriksson, Johan Dahlstrand, Peter Friberg

**Affiliations:** 1 School of Public Health and Community Medicine, University of Gothenburg, Sweden; 2 Centre for Psychiatry Research, Karolinska Institutet and Stockholm Health Care Services, Sweden; 3 Department of Data and Analysis, Region Västra Götaland, Sweden; 4 The Swedish Institute for Global Health Transformation (SIGHT), Royal Swedish Academy of Sciences, Sweden

**Keywords:** Adolescents, COVID-19, psychosomatic symptoms, stress, happiness, health behaviours, longitudinal

## Abstract

Aims: There is an urgent need to explore the impact of the COVID-19 pandemic on adolescent mental health and health behaviours. To date, there are no such studies on Swedish adolescents. As COVID-19 emerged in the middle of our ongoing 2-year follow-up examination of the Study of Adolescence Resilience and Stress, we had the unique opportunity to use the corona outbreak as a ‘natural experiment’ to study the impact of COVID-19 on 15-year-old adolescents in Sweden. Methods: Adolescents (baseline age 13.6±0.4 years) were recruited from schools in western Sweden (during the COVID-19 outbreak schools were kept open for those under 16 years of age). The COVID-19 pandemic reached Sweden on 31 January 2020. A total of 1316 adolescents answered the 2-year follow-up survey before (unexposed to COVID-19 pandemic, controls) and 584 after 1 February 2020 (COVID19-exposed). Data on stress, psychosomatic symptoms, happiness, relationships with parents and peers, school and health behaviours were collected. Results: Adolescents reported higher levels of stress and psychosomatic symptoms and lower levels of happiness at follow-up compared to baseline. These changes occurred to a similar extent in both the control and COVID-19-exposed groups. Likewise, the COVID-19-exposed group showed no deterioration in peer relations or relations with parents versus controls. We did not find any significant differences between groups regarding sleep duration and physical activity. **Conclusions: Swedish adolescents exposed to COVID-19 during most of 2020 showed no differences in longitudinal changes in mental health, relationships with parents and peers, and health behaviours compared to those not exposed to COVID-19**.

## Background

The COVID-19 pandemic imposes a massive change to the psychosocial environment in affected countries. There is an urgent need to explore the effects of this pandemic on the mental health in adolescents [1, 2].

Data regarding the COVID-19 pandemic’s impact on adolescents are evolving [3]. There are studies reporting a high prevalence of anxiety and depressive symptoms, increased loneliness, perceived changes in relationships with family and friends [4–7]. Impacts of COVID-19 on children and adolescents’ health behaviour have also been reported, such that children and youth have had decreased physical activity levels, increased sedentary behaviour and increased sleep during the outbreak [8–10].

Unlike many other nations, Sweden has kept its schools open for those under 16 years of age during the early phase of the COVID-19 outbreak [11]. Sweden has implemented physical distancing, rather than enforcing lockdown, to minimise the spread of COVID-19 [12]. To date, there is no published study on the impact of COVID-19 on mental health and health behaviour in Swedish adolescents.

The aim of this study was hence to investigate the impacts of COVID-19 on mental health, perceived relationships with parents and peers and health behaviours in 15-year-old adolescents in Sweden. We used data collected from our ongoing longitudinal Study of Adolescence Resilience and Stress (STARS). The COVID-19 pandemic was confirmed to have reached Sweden on 31 January 2020, at the same time as the World Health Organization (WHO) declared a public health emergency of international concern [11]. Given that COVID-19 emerged in the middle of our ongoing 2-year follow-up examination, we had the unique opportunity, using the prevailing situation as a ‘natural experiment’ in studying the initial effects of COVID-19 on adolescents.

The advantages are two-fold. First, we had pre-COVID-19 comparative baseline data characterising all adolescents. Only longitudinal studies that include a baseline measure before COVID-19 can truly detect changes related to the COVID-19 pandemic, and studies with pre-COVID-19 data are few [13]. Second, as mental health problems are known to increase from early adolescence onwards [14], it is important to ensure that the potential changes in mental health problems during ongoing COVID-19 are not simply due to such age-related effects. In this respect, we were able to study the effects of COVID-19 by comparing adolescents with or without COVID-19 exposure.

## Methods

We used data from the baseline and 2-year follow-up surveys of the STARS. At baseline, participants were 7th grade pupils recruited from 54 schools in 16 municipalities in western Sweden. A total of 2283 pupils (age 13.6±0.4 years, 44% boys) were examined during September 2015 and June 2019. A total of 1900 adolescents participated in the 2-year follow-up survey, divided into control (re-examined during September 2017 and January 2020) and COVID-19-exposed (re-examined during February and November 2020). The time between the two waves was 2.0±0.2 (range 0.8–2.6) and 2.0±0.1 (range 1.4–2.6) years for the control and COVID-19-exposed groups, respectively. The study was approved by the ethics committee at the Sahlgrenska Academy, University of Gothenburg, and written informed consents were obtained.

Adolescents answered a web questionnaire regarding stress [15], psychosomatic symptoms [16], happiness [17], relations with parents and home life [18], social support and peers [18], school environment [18], sleep duration and physical activity [19], feeling in general and belief in the future. Data on gender and migration background were collected. Family socioeconomic status (SES) was measured using the family affluence scale [20].

The paired Student’s *t*-test was used for analysing differences between baseline and 2-year follow-up. General linear models (GLMs) for repeated measures were used to examine group differences in repeated measure effects. SPSS 27 was used for statistical analyses. *P*⩽0.05 was considered statistically significant.

## Results

There was no significant difference between the two groups regarding sex (control: 42.7% boys, COVID-19-exposed: 46.8% boys) and family SES (control: 26.3%/58.7%/15%; COVID-19-exposed: 25%/62.6%/12.5%, low/medium/high SES, respectively). The percentage of adolescents with migrant background was lower in the COVID-19-exposed than the control group (14.6% vs. 22.5%, *P*<0.001, chi-square test).

Compared to baseline, levels of perceived stress and psychosomatic symptoms increased, while levels of happiness, peer relations, relations with parents, school satisfaction, sleep hours and days with at least 60 minutes moderate-to-vigorous physical activity per week decreased at the 2-year follow-up (Table I). No changes in leisure-time exercise were found.

**Table I.** Baseline and 2-year follow-up measures in adolescents, and differences in repeated measure effects between control and COVID-19-exposed group.

	**Control**				**COVID-19-exposed**				**GLMs for repeated measures**	
	*N*	Baseline	2-year follow-up	*P* value	*N*	Baseline	2-year follow-up	*P* value	*P* for repeated measure effect	*P* for group difference
Age (years)	1317	13.5±0.4 (11.6–16.1)	15.5±0.4 (13.7–17.7)		584	15.5±0.4 (14.5–18.0)	15.5±0.4 (14.5–18.0)			
Stress	1306	15.1±5.9	16.7±6.4	<0.001	577	15.8±6.3	17.1±6.3	<0.001	<0.001	0.424
Psychosomatic symptoms	1306	11.2±5.4	11.8±6.0	<0.001	577	12.0±5.7	12.1±6.2	0.674	0.007	0.048
Happiness	1308	30.5±6.3	28.2±6.9	<0.001	580	29.6±7.1	27.2±7.5	<0.001	<0.001	0.551
Peer relations	1311	16.2±2.9	15.9±3.4	<0.001	582	16.1±3.4	15.8±3.5	0.076	<0.001	0.794
Relations with parents	1304	20.8±3.3	20.3±3.9	<0.001	580	20.9±3.5	20.3±3.8	<0.001	<0.001	0.667
School satisfaction	1307	11.2±1.9	10.9±2.2	<0.001	579	11.1±2.0	10.6±2.2	<0.001	<0.001	0.11
Sleep hours on a school day	1313	8.58±0.85	7.99±0.95	<0.001	582	8.50±0.86	7.97±0.95	<0.001	<0.001	0.241
Sleep hours on a non-school day	1313	9.85±1.25	9.50±1.26	<0.001	582	9.84±1.29	9.59±1.25	<0.001	<0.001	0.162
Physical activity 60 min/day (days/week)	1315	3.9±1.8	3.9±1.9	0.002	583	4.1±1.7	3.9±1.9	0.049	0.033	0.453
Weekly duration of leisure-time exercise	1315	4.1±1.4	4.1±1.5	0.954	583	4.1±1.4	4.1±1.5	0.201	0.312	0.283
Feeling in general	1316	8.1±1.6	7.4±1.9	<0.001	584	7.8±1.7	7.3±1.9	<0.001	<0.001	0.256
Belief in the future	1300	8.3±1.4	8.3±1.6	0.363	573	8.3±1.6	8.1±1.7	0.077	0.044	0.305

Variables are presented as mean±SD.

The paired Student’s *t*-test was used for analysing differences between the baseline and 2-year follow-up.

General linear models (GLMs) for repeated measures were used to examine group differences in repeated measure effects.

Analysing group-related differences in repeated measure effects over the 2-year period, we did not find any statistically significant difference between the control and COVID-19-exposed groups in the variables examined, except one, namely psychosomatic symptoms. The level of psychosomatic symptoms increased over the 2-year period in the control but not in the COVID-19-exposed group (Table I).

Given that sex-related differences were observed in almost all variables (data not shown), we did GLM analysis with sexes separated. The changes over the 2-year period were similar among boys (Table II) and girls (Table III), except psychosomatic symptoms, with boys reporting a decreased level and girls reporting an increased level over the 2-year period, and the days with at least 60 minutes physical activity per week being significantly decreased in girls but not in boys.

**Table II.** Baseline and 2-year follow-up measures in male adolescents, and differences in repeated measure effects between control and COVID-19-exposed group.

	**Control**				**COVID-19-exposed**				**GLMs for repeated measures**	
	*N*	Baseline	2-year follow-up	*P* value	*N*	Baseline	2-year follow-up	*P* value	*P* for repeated measure effect	*P* for group difference
Age (years)	565	13.5±0.4 (11.6–14.8)	15.5±0.4 (13.7–16.6)		273	13.5±0.4 (12.7–14.9)	15.5±0.4 (14.5–16.9)			
Stress	558	13.5±5.6	14.3±5.8	0.001	270	14.0±5.9	14.5±5.7	0.188	0.003	0.689
Psychosomatic symptoms	558	9.5±5.0	9.1±5.2	0.071	270	10.0±5.1	9.0±5.5	0.003	<0.001	0.115
Happiness	560	32.1±5.3	30.7±6.0	<0.001	271	31.4±6.7	29.9±6.8	<0.001	<0.001	0.818
Peer relations	561	15.9±2.8	15.5±3.4	0.003	272	15.8±3.2	15.6±3.6	0.234	0.007	0.475
Relations with parents	558	21.2±3.0	20.9±3.6	0.043	270	21.2±3.4	20.9±3.7	0.148	0.018	0.914
School satisfaction	559	11.1±1.9	10.8±2.3	0.006	270	11.0±2.0	10.6±2.3	0.013	<0.001	0.651
Sleep hours on a school day	563	8.73±0.75	8.08±0.96	<0.001	272	8.68±0.79	8.16±0.91	<0.001	<0.001	0.066
Sleep hours on a non-school day	563	9.81±1.27	9.48±1.29	<0.001	272	9.82±1.33	9.50±1.18	<0.001	<0.001	0.974
Physical activity 60min/day (days/week)	564	4.3±1.8	4.1±1.9	0.09	273	4.2±1.7	4.3±1.9	0.683	0.53	0.189
Weekly duration of leisure-time exercise	564	4.3±1.3	4.3±1.4	0.63	273	4.2±1.3	4.3±1.5	0.05	0.058	0.176
Feeling in general	564	8.5±1.4	8.0±1.7	<0.001	273	8.3±1.6	8.0±1.7	0.001	<0.001	0.426
Belief in the future	557	8.4±1.3	8.5±1.6	0.44	268	8.3±1.7	8.5±1.5	0.218	0.15	0.585

Variables are presented as mean±SD.

The paired Student’s *t*-test was used for analysing differences between the baseline and 2-year follow-up.

General linear models (GLMs) for repeated measures were used to examine group differences in repeated measure effects.

**Table III.** Baseline and 2-year follow-up measures in female adolescents, and differences in repeated measure effects between control and COVID-19-exposed group.

	**Control**				**COVID-19-exposed**				**GLMs for repeated measures**	
	*N*	Baseline	2-year follow-up	*P* value	*N*	Baseline	2-year follow-up	*P* value	*P* for repeated measure effect	*P* for group difference
Age (years)	752	13.6±0.4 (12.0–16.1)	15.5±0.4 (14.3–17.7)		311	13.5±0.4 (12.3–16.0)	15.5±0.4 (14.5–18.0)			
Stress	748	16.3±5.9	18.4±6.3	<0.001	307	17.4±6.2	19.4±5.8	<0.001	<0.001	0.907
Psychosomatic symptoms	748	12.4±5.4	13.8±5.7	<0.001	307	13.8±5.5	14.8±5.5	0.001	<0.001	0.366
Happiness	748	29.3±6.7	26.4±6.9	<0.001	309	28.1±7.1	24.8±7.4	<0.001	<0.001	0.411
Peer relations	750	16.5±3.0	16.2±3.3	0.037	310	16.3±3.6	16.0±3.4	0.186	0.02	0.806
Relations with parents	746	20.6±3.6	19.9±4.0	<0.001	310	20.7±3.5	19.9±3.9	<0.001	<0.001	0.564
School satisfaction	748	11.3±1.9	11.0±2.1	<0.001	309	11.2±2.1	10.6±2.1	<0.001	<0.001	0.069
Sleep hours on a school day	750	8.47±0.89	7.92±0.94	<0.001	310	8.35±0.88	7.80±0.96	<0.001	<0.001	0.989
Sleep hours on a non-school day	750	9.89±1.22	9.52±1.23	<0.001	310	9.85±1.25	9.66±1.30	0.014	<0.001	0.054
Physical activity 60min/day (days/week)	751	3.7±1.7	3.7±1.9	0.858	310	3.9±1.8	3.6±1.8	0.01	0.015	0.025
Weekly duration of leisure-time exercise	751	4.0±1.4	3.9±1.5	0.635	310	4.0±1.4	4.0±1.5	0.90	0.718	0.872
Feeling in general	752	7.8±1.6	7.0±1.9	<0.001	311	7.4±1.6	6.6±1.9	<0.001	<0.001	0.537
Belief in the future	743	8.2±1.5	8.1±1.6	0.056	305	8.2±1.5	7.8±1.7	0.001	<0.001	0.041

Variables are presented as mean±SD.

The paired Student’s *t*-test was used for analysing differences between the baseline and 2-year follow-up.

General linear models (GLMs) for repeated measures were used to examine group differences in repeated measure effects.

The group-related difference in psychosomatic symptoms disappeared when we analysed boys and girls separately (Tables II and III). On the other hand, we observed group-related differences in the days with at least 60 minutes moderate-to-vigorous physical activity per week and belief in the future in girls but not boys, both variables decreased significantly in the COVID-19-exposed group but not in controls.

Changes of the variables over the 2-year period did not differ with respect to time of COVID-19 exposure (see Supplemental material).

## Discussion

Using longitudinal data collected in the STARS cohort, we found that adolescents reported higher levels of stress and psychosomatic symptoms and lower levels of happiness from 13 to 15 years of age. These changes occurred in both the controls and COVID-19-exposed groups, with no differences between the groups, suggesting that the changes are age related rather than COVID-19 related.

The present results contrast with previous studies showing that adolescents experienced an increase in depressive symptoms, anxiety and loneliness and a decrease in life satisfaction compared to pre-COVID-19 measures [6, 7]. These studies were performed in countries where schools were temporarily closed in an attempt to contain the spread of the COVID-19 pandemic. The most distressing issue for adolescents during COVID-19 was not being able to see their friends, and perceived social changes were associated with an increase in mental health problems [7]. Adolescents were more concerned about governmental restrictions designed to contain the spread of the virus than the virus itself [7]. Furthermore, experiencing difficulties with online learning significantly moderated changes in depressive symptoms.

From February to November, the period when we collected data for the COVID-19-exposed group, Sweden kept its schools open for those under 16 years of age. This may have helped adolescents to keep a relatively ‘normal’ life despite the ongoing pandemic. This notion is supported by our observations that adolescents in the COVID-19-exposed group did not experience worse changes in peer relations than those in the control group. Furthermore, there were no significant differences between the control and the COVID-19-exposed group regarding sleep duration and physical activity, suggesting that adolescents in the COVID-19-exposed group could keep their daily routines during the pandemic.

## Conclusions

Swedish adolescents exposed to COVID-19 showed no differences in longitudinal changes in mental health, relationships with parents and peers, and health behaviours as compared to those not exposed to COVID-19. This may be explained by the Swedish authorities’ decision to keep schools open to this age group.

## Supplemental Material

sj-docx-1-sjp-10.1177_14034948211021724 – Supplemental material for Impact of COVID-19 pandemic on mental health and health behaviors in Swedish adolescents Click here for additional data file. Supplemental material, sj-docx-1-sjp-10.1177_14034948211021724 for Impact of COVID-19 pandemic on mental health and health behaviors in Swedish adolescents by Yun Chen, Walter Osika, Göran Henriksson, Johan Dahlstrand and Peter Friberg in Scandinavian Journal of Public Health

## References

[1] Holmes EA O’Connor RC Perry VH , et al. Multidisciplinary research priorities for the COVID-19 pandemic: a call for action for mental health science. Lancet Psychiatry 2020;7:547–560. 32304649 10.1016/S2215-0366(20)30168-1 PMC7159850

[2] Jansen D Kosola S Arevalo LC , et al. Child and adolescent health needs attention now, and in the aftermath of the COVID-19 pandemic. Int J Public Health 2020;65:723–725. 32740686 10.1007/s00038-020-01446-8 PMC7395573

[3] Singh S Roy D Sinha K , et al. Impact of COVID-19 and lockdown on mental health of children and adolescents: a narrative review with recommendations. Psychiat Res 2020;293:113429. 10.1016/j.psychres.2020.113429 PMC7444649 32882598

[4] Duan L Shao X Wang Y , et al. An investigation of mental health status of children and adolescents in china during the outbreak of COVID-19. J Affect Disord 2020;275:112–118. 32658812 10.1016/j.jad.2020.06.029 PMC7329661

[5] Zhou SJ Zhang LG Wang LL , et al. Prevalence and socio-demographic correlates of psychological health problems in Chinese adolescents during the outbreak of COVID-19. Eur Child Adolesc Psychiatry 2020;29:749–758. 32363492 10.1007/s00787-020-01541-4 PMC7196181

[6] Rogers AA Ha T Ockey S. Adolescents’ perceived socio-emotional impact of COVID-19 and implications for mental health: results from a U.S.-based mixed-methods study. J Adolesc Health 2021, 68 (1): 43–52. 10.1016/j.jadohealth.2020.09.039 PMC7605752 33143986

[7] Magson NR Freeman JYA Rapee RM , et al. Risk and protective factors for prospective changes in adolescent mental health during the COVID-19 pandemic. J Youth Adolesc 2021; 50: 44–57. 10.1007/s10964-020-01332-9 PMC7590912 33108542

[8] Xiang M Zhang Z Kuwahara K. Impact of COVID-19 pandemic on children and adolescents’ lifestyle behavior larger than expected. Prog Cardiovasc Dis 2020;63:531–532. 32360513 10.1016/j.pcad.2020.04.013 PMC7190470

[9] Moore SA Faulkner G Rhodes RE , et al. Impact of the COVID-19 virus outbreak on movement and play behaviours of Canadian children and youth: a national survey. Int J Behav Nutr Phy 2020;17:85. 10.1186/s12966-020-00987-8 PMC7336091 32631350

[10] Sekulic D Blazevic M Gilic B , et al. Prospective analysis of levels and correlates of physical activity during COVID-19 pandemic and imposed rules of social distancing; Gender specific study among adolescents from Southern Croatia. Sustainability-Basel 2020;12:4072.

[11] Ludvigsson JF. The first eight months of Sweden’s COVID-19 strategy and the key actions and actors that were involved. Acta Paediatr 2020;109:2459–2471. 32951258 10.1111/apa.15582 PMC7537539

[12] Kavaliunas A Ocaya P Mumper J , et al. Swedish policy analysis for Covid-19. Health Policy Techn 2020;9:598–612. 10.1016/j.hlpt.2020.08.009 PMC7455549 32904437

[13] Racine N Cooke JE Eirich R , et al. Child and adolescent mental illness during COVID-19: a rapid review. Psychiatry Res 2020;292:113307. 32707216 10.1016/j.psychres.2020.113307 PMC7363598

[14] Kessler RC Berglund P Demler O , et al. Lifetime prevalence and age-of-onset distributions of DSM-IV disorders in the National Comorbidity Survey Replication. Arch Gen Psychiatry 2005;62:593–602. 15939837 10.1001/archpsyc.62.6.593

[15] Cohen S Deneise J-D. Who’s stressed? Distribution of psychological stress in the United States in probability samples from 1983, 2006, and 2009. J Appl Soc Psychol 2012;42:1320–1334.

[16] Friberg P Hagquist C Osika W. Self-perceived psychosomatic health in Swedish children, adolescents and young adults: an internet-based survey over time. BMJ Open 2012;2:e000681. 10.1136/bmjopen-2011-000681 PMC4400616 22855621

[17] Hills P Argyle M. The Oxford happiness questionnaire: a compact scale for the measurement of psychological well-being. Personal Individ Differences 2002;33:1073–1082.

[18] Ravens-Sieberer U Gosch A Rajmil L , et al. The KIDSCREEN-52 quality of life measure for children and adolescents: psychometric results from a cross-cultural survey in 13 European countries. Value Health: the journal of the International Society for Pharmacoeconomics and Outcomes Research 2008;11:645–658. 10.1111/j.1524-4733.2007.00291.x 18179669

[19] Kalman M Inchley J Sigmundova D , et al. Secular trends in moderate-to-vigorous physical activity in 32 countries from 2002 to 2010: a cross-national perspective. Eur J Public Health 2015;25(Suppl. 2):37–40. 25805785 10.1093/eurpub/ckv024

[20] Torsheim T Cavallo F Levin KA , et al.; Group FASDS: Psychometric Validation of the Revised Family Affluence Scale: a latent variable approach. Child Indic Res 2016;9:771–784. 27489572 10.1007/s12187-015-9339-x PMC4958120

