# Supplementary material for: Impact of COVID-19 pandemic on mental health and health behaviors in Swedish adolescents
Source: Scand J Public Health. 2021 Jun 8;50(1):26–32. doi: 10.1177/14034948211021724 (PMC8808000; doi:10.1177/14034948211021724)
Supplement: sj-docx-1-sjp-10.1177_14034948211021724 – Supplemental material for Impact of COVID-19 pandemic on mental health and health behaviors in Swedish adolescents [file sj-docx-1-sjp-10.1177_14034948211021724.docx]

Supplementary material

Questionnaire:

Stress was measured using the 10-item version of the perceived stress scale (PSS-10) (1). Adolescents were asked to rate each statement with a 5-point frequency scale that ranges from 0 (never) to 4 (very often). A maximum of one missing item was accepted and missing item was replaced by intrapersonal mean. The total score ranged from 0 to 40, with higher score representing higher level of stress. In our samples, the Cronbach’s alpha is 0.812.

The psychosomatic problem scale was used to measure psychosomatic symptoms including difficulty in concentrating and sleeping, suffering from headaches and stomach-aches, poor appetite, feeling tense, low and dizzy (2). Participants were asked to choose between 0 (never) and 4 (always). A maximum of one missing item was accepted and missing item was replaced by intrapersonal mean before a total score was calculated. The total score ranged from 0 to 32, with higher score representing higher level of psychosomatic symptoms. In our samples, the Cronbach’s alpha is 0.820.

Happiness was assessed using the 8-item Oxford Happiness Questionnaire (OHQ) developed by Hills and Argyle (3). The OHQ employs a 6-point Likert scale response format from strongly disagree = 0 to strongly agree = 5. A maximum of one missing item was accepted and missing item was replaced by intrapersonal mean. The total score ranged from 0 to 40, with higher scores corresponding to higher levels of happiness. In our samples, the Cronbach’s alpha is 0.895.

Relation with parents and home life were measured using 6 questions, such as “Did you feel good at home?”, “Did you think your parents understand you?”, “Did they have enough time for you?” (4). Participants were asked to choose between 0 (never) and 4 (always). The total score ranged from 0 to 24. In our sample, the Cronbach’s alpha is 0.872.

Social support and peers were measured using 5 questions, such as “Were you with your friends?”, “Were you able to get support from your friends?” (4). Participants were asked to choose between 0 (never) and 4 (always). The total score ranged from 0 to 20. In our sample, the Cronbach’s alpha is 0.798.

School environment was measured using 3 questions, such as “Did you have a good time at school?” (4). Participants were asked to choose between 0 (never) and 4 (always). The total score ranged from 0 to 12. In our sample, the Cronbach’s alpha is 0.724.

Participants answered questions about bedtime and rise time on a school day and a non-school day. Sleep duration was calculated by estimating the time between bedtime and rise time.

The WHO HBSC physical activity questionnaire was used to measure the number of days over the past week during which adolescents were physically active for a total of at least 60 minutes (5).

Feeling in general and belief in the future were measured using single item questions with score ranging from 1 (low) to 10 (high).

Family socioeconomic status (SES) was measured using Family Affluence Scale with 6 questions regarding car ownership, own bedroom, computer ownership, number of bathrooms, dishwasher and number of holidays abroad (6).

Results

Figure supplementary. Changes of perceived stress (a) and psychosomatic symptoms (b) over the 2-year period in the control group and the COVID19-exposed group.


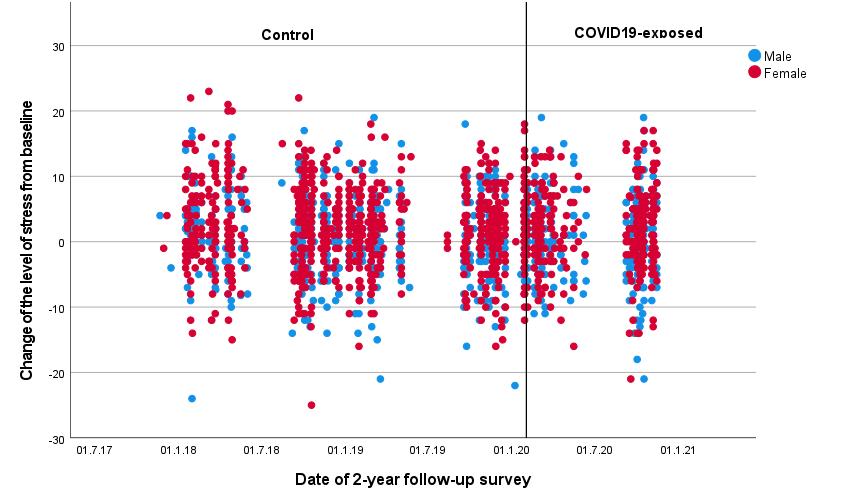
a)

b)


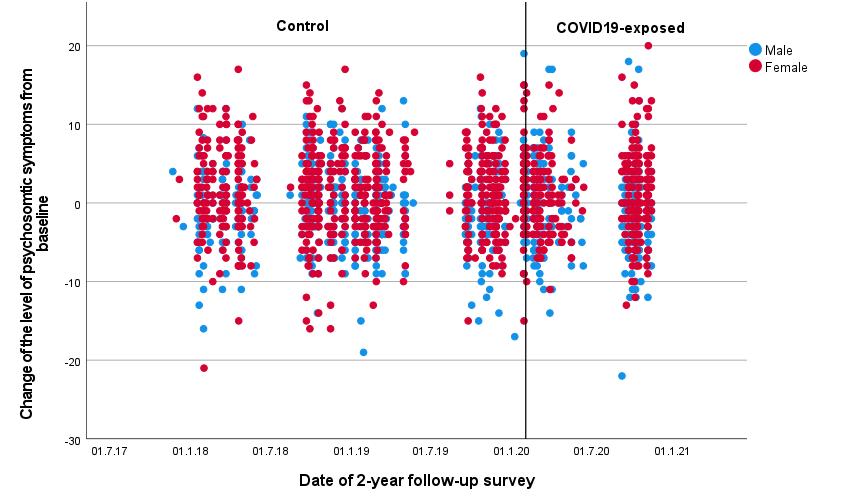


Figure supplementary

References

1. Cohen S*, et al.* (2012) Who's Stressed? Distributions of Psychological Stress in the United States in Probability Samples from 1983, 2006, and 2009. *J Appl Soc Psychol* 42(6):1320-1334.

2. Friberg P*, et al.* (2012) Self-perceived psychosomatic health in Swedish children, adolescents and young adults: an internet-based survey over time. *BMJ open* 2(4).

3. Hills P*, et al.* (2002) The Oxford happiness questionnaire: a compact scale for the measurement of psychological well-being. *Personality and Individual Differences* 33:1073-1082.

4. Ravens-Sieberer U*, et al.* (2008) The KIDSCREEN-52 quality of life measure for children and adolescents: psychometric results from a cross-cultural survey in 13 European countries. *Value in health : the journal of the International Society for Pharmacoeconomics and Outcomes Research* 11(4):645-658.

5. Kalman M*, et al.* (2015) Secular trends in moderate-to-vigorous physical activity in 32 countries from 2002 to 2010: a cross-national perspective. *Eur J Public Health* 25 Suppl 2:37-40.

6. Torsheim T*, et al.* (2016) Psychometric Validation of the Revised Family Affluence Scale: a Latent Variable Approach. *Child Indic Res* 9:771-784.
